# Ecological traits for 1374 arthropod species collected in a German grassland

**DOI:** 10.1002/ecy.70077

**Published:** 2025-04-23

**Authors:** Maximilian Bröcher, Sebastian T. Meyer, Ana Garcia Leher, Anne Ebeling

**Affiliations:** ^1^ Population Ecology Group, Institute of Biodiversity, Ecology and Evolution, University of Jena Jena Germany; ^2^ Terrestrial Ecology Research Group School of Life Sciences, Technical University of Munich Freising Germany

**Keywords:** Arachnida, body length, body mass, Coleoptera, effect trait, feeding ecology, flight capability, habitat requirements, Hemiptera, Hymenoptera, Jena experiment, response trait

## Abstract

Arthropods play an important role in grasslands, making trait‐based research a valuable approach to advance our understanding of ecosystem functioning. However, a wide range of functional traits for complex arthropod communities is often not available in a single source but must be compiled from multiple references and databases. Using suction and pitfall sampling in the field site of the Jena Experiment, we collected Araneae, Coleoptera, Hemiptera, Hymenoptera, Isopoda, Myriapoda, and Orthoptera over a period of 10 years to document arthropod taxa in the area. We then surveyed the existing literature to compile nine important functional traits for each species. The nine selected traits cover information about feeding ecology (feeding guild, feeding source, food acquisition, feeding mode, food specialization), habitat requirements (stratum), flight capability (aerial mobility), and size (body mass, body length). As the selected traits cover both response traits and effect traits, this database can be deployed for investigations on topics ranging from the sensitivity of arthropod communities to environmental changes (response traits) to the impact of arthropods on the functioning of ecosystems (effect traits). There are no copyright constraints associated with the use of the data, except for the citing of this Data Paper.

## CONFLICT OF INTEREST STATEMENT

The authors declare no conflicts of interest.

## Supporting information


Appendix S1:

## Data Availability

The dataset is available as Supporting Information. Data are also available in the Jena Experiment Information System (JEXIS) repository at https://doi.org/10.25829/Q570-JR82.

