# Supplementary material for: Ecological traits for 1374 arthropod species collected in a German grassland
Source: Ecology. 2025 Apr 23;106(4):e70077. doi: 10.1002/ecy.70077 (PMC12015999; doi:10.1002/ecy.70077)
Supplement: Supplementary file 1 — Appendix S1: [file ECY-106-e70077-s001.zip › DataS1/MetadataS1.pdf]

Data Paper published in *Ecology*, an Ecological Society of America journal

Metadata S1

Ecological traits for 1,374 arthropod species collected in a German grassland

Maximilian Bröcher, Sebastian T. Meyer, Ana Garcia Leher, Anne Ebeling

We have ensured that the dataset presented in this paper has been thoroughly documented, including detailed descriptions of data collection methods and processing protocols. Data are provided as supporting information with this data paper and are archived in Bröcher et al. (2025) in The Jena Experiment Information System at https://doi.org/10.25829/Q570-JR82. We have included all relevant metadata in this document and in the repository. We have adhered to all ethical guidelines and best practices in the collection, preparation and sharing of our data. The integrity of the data has been maintained through rigorous quality control measures, and any limitations or uncertainties have been transparently reported. We encourage collaboration and reuse of our data by the broader ecological community and welcome researchers to use this data in new and innovative ways. Any feedback, questions, or collaborative proposals can be directed to: Anne Ebeling: Institute of Ecology and Evolution, University Jena, Dornburger Strasse 159, D-07743 Jena, Germany. E-mail: anne.ebeling@uni-jena.de.

Class I. Data Set Descriptors

A. Data set identity: Functional traits of grassland arthropods

B. Data set identification code:

species_traits_JE.csv

C. Data set description

1. Originators:

Anne Ebeling: Institute of Ecology and Evolution, University Jena, Dornburger Strasse 159, D-07743 Jena, Germany. E-mail: anne.ebeling@uni-jena.de

Sebastian T. Meyer: Terrestrial Ecology Research Group, School of Life Sciences, Technical University of Munich, Hans-Carl-von-Carlowitz-Platz 2, D-85354 Freising, Germany.

2. Abstract: Arthropods play an important role in grasslands, making trait-based research a valuable approach to advance our understanding of ecosystem functioning. However, a wide range of functional traits for complex arthropod communities is often not available in a single source, but must be compiled from multiple references and databases. Using suction and pitfall sampling in the field site of the Jena Experiment, we collected 1,374 arthropod species (Arachnida, Coleoptera, Hemiptera, Hymenoptera, Isopoda, Myriapoda, and Orthoptera) over a period of 10 years to document arthropod taxa in the area. We then surveyed existing literature to compile nine important functional traits for each species. The nine selected traits give information about feeding ecology (feeding guild, feeding source, food acquisition, feeding mode, food specialization), habitat requirements (stratum), flight capability (aerial mobility), and size (body mass, body length). As the selected traits cover both response traits and effect traits, this database can be deployed for

investigations on topics ranging from the sensitivity of arthropod communities to environmental changes (response traits) to the impact of arthropods on the functioning of ecosystems (effect traits). There are no constraints associated with the use of the data, except citing of this Data Paper.

D. Key words/phrases: *Arachnida, Coleoptera, Hemiptera, Hymenoptera, feeding ecology, habitat requirements, flight capability, body mass, body length, response trait, effect trait, Jena Experiment*

Class II. Research origin descriptors

A. Overall project description:

1. **Identity:** Functional traits of 1,374 arthropod species occurring in European grasslands

2. **Originators:**

Anne Ebeling: Institute of Ecology and Evolution, University Jena, Dornburger Strasse 159, D-07743 Jena, Germany. E-mail: anne.ebeling@uni-jena.de

Sebastian T. Meyer: Terrestrial Ecology Research Group, School of Life Sciences, Technical University of Munich, Hans-Carl-von-Carlowitz-Platz 2, D-85354 Freising, Germany. E-mail: sebastian.t.meyer@tum.de

3. **Period of study:** 2010-2020

4. **Objectives:**

In times of global change, it is more important than ever before to predict how ecosystems will respond to altered environmental conditions. For a long time, ecological research has quantified ecosystem response based on measures of biodiversity (e.g. species richness, abundance), as they are easy to measure and often strong predictors of multiple ecosystem factors. However, they lack the depth to fully capture ecosystem complexity and functioning (McGill *et al.*, 2006; Wong, Guénard and Lewis, 2019). Trait-based research bridges this gap by linking biodiversity to ecosystem processes, enabling a deeper understanding of ecological dynamics and more accurate predictions of ecosystem responses to environmental changes (Díaz and Cabido, 2001; McGill *et al.*, 2006; Violle *et al.*, 2007; Lavorel *et al.*, 2013; Bello *et al.*, 2021). Arthropods hold important ecosystem functions, as herbivory, pest control, pollination or decomposition (Noriega *et al.*, 2018; Schowalter, Noriega and Tscharntke, 2018). Depending on the set of traits that arthropod species possess, they (1) react differently to environmental changes and (2) the impact they have on the ecosystem varies in strength (Martínez-Núñez *et al.*, 2024). Consequently, it is essential to incorporate functional traits of arthropod species into ecological research.

Arthropods are a diverse group of organisms and getting trait information for all species occurring in an ecosystem is difficult to impossible. Existing databases usually provide only a few traits or do not cover many species, so that information has to be compiled from a variety of individual sources. To simplify this time-consuming work for future studies, we have compiled nine functional traits from literature (feeding guild, feeding source, food acquisition, feeding mode, food specialization, stratum, aerial mobility, body mass, body length; Table 1) for 1,374 species occurring in an Arrhenatheretum grassland community in central Germany.

Feeding guild, feeding source, food acquisition, feeding mode and food specialization give information on the type of food resource that is consumed by the species, the energetic expenditures associated with finding and consuming their resource and their flexibility in food resources they can cope with. Stratum describes the habitat layer inhabited by the taxa and aerial mobility about their flight capability (indication for e.g. dispersal ability). Body mass, calculated from body length, is considered a to be a “super trait”, as it is integrally linked to a wide array of biological, ecological, and evolutionary processes (Brose *et al.*, 2006). It’s ability to predict and influence multiple other traits and interactions makes it a cornerstone in the study of life sciences, underscoring its significance in understanding organismal biology and ecosystem dynamics. This central role allows body mass to provide valuable insights into species mobility, resource use, and space use (Woodward *et al.*, 2005). The data set supports future research on how environmental changes impact the functioning of grassland ecosystems.

5. Abstract:

Arthropod consumers contribute to important ecosystems processes such as herbivory, pest control, and decomposition, with their responses to environmental changes varying based on their functional traits. Recognizing the challenge of acquiring trait information for complex arthropod communities, we compiled nine functional traits from literature for 1,374 grassland species in central Germany. These traits are feeding guild, feeding source, food acquisition, feeding mode, food specialization, stratum, aerial mobility, body mass and body length. The selected traits provide insights into species' feeding ecology, habitat

preferences, and ecological roles, facilitating more comprehensive ecological research and informing predictions about ecosystem responses to global change.

6. Sources of funding:

DFG FOR 1451 EB 555/3-1, HI 1941/1-1, WE 3081/15-1, WE 3081/15-2

DFG FOR 5000 EB 555/6-1, EB 555/6-2, ME 5474/1-1, ME 5474/1-2

B. Specific subproject description

1. Site description:

The arthropod species presented in this dataset come from regular sampling campaigns conducted in the framework of the Jena Experiment, a large biodiversity experiment established in 2002. The 10-ha field site is located in Jena, Germany (50°55'N, 11°35'E; 130m a. s. l.) and since the start of the experiment managed as an extensively-used hay meadow (Roscher *et al.*, 2004). The mean annual air temperature and precipitation, calculated from climate data between 2004 and 2021, was 9.8°C and 571 mm, respectively (Huang *et al.*, 2024). The soil of the field site is classified as an Eutric Fluvisol (FAO-Unesco 1997), formed from loamy fluvial sediments reaching up to 2 meters in thickness, and nearly free of stones. The target plant community on the experimental field site is a semi-natural species-rich mesophilic grassland, belonging to the Molinio-Arrhenatheretea meadows (Roscher *et al.*, 2004; Ellenberg and Leuschner, 2010). Managed as hay meadow, the field site is mown twice a year at the maximum standing biomass in early June and September.

2. Experimental or sampling design:

Data collection was done in the Main Experiment (80 plots of $100m^2$ size, differing in plant species richness from 1 to 60 species; Roscher *et al.*, 2004), and the Trait-Based Experiment (138 plots of $12.25m^2$ size, differing in plant species richness from 1-8; Ebeling *et al.*, 2014). Ground- and vegetation-associated arthropods were sampled in ten years (2010, 2012, 2013, 2014, 2015, 2016, 2017, 2018, 2019, 2020) using pitfall traps (Main Experiment 42 sampling dates, Trait-Based Experiment 37 sampling dates) and suction sampling (Main Experiment 23 sampling dates, Trait-Based Experiment 11 sampling dates). Pitfall traps were of 4.5 cm in size and emptied every two weeks (except during the mowing periods). Suction sampling was conducted on sunny days between 9 am and 4 pm in randomly placed cages of $0.75 m^3$, using a modified commercial vacuum cleaner (Kärcher A2500, Kärcher GmbH, Winnenden, Germany). All sampled arthropods were stored in 70% ethanol before further processing. After sorting into taxonomic groups (Arachnida, Coleoptera, Hemiptera, Hymenoptera, Isopoda, Myriapoda, and Orthoptera), individuals were sent to the following experts for identification: Roland Achtziger (Auchenorrhyncha), Eric Anton (Coleoptera), Theo Blick (Araneae), Frank Creutzburg (Hymenoptera), Ralf Heckmann (Heteroptera), Günter Köhler (Orthoptera), Gerlinde Kratzsch (Isopoda, Diplopoda, Myriapoda), Norman Lindner (Isopoda), Michael Meyer (Myriapoda), Christoph Muster (Araneae, Opiliones), Franz Schmolke (Heteroptera), Oliver Wiche (Auchenorrhyncha). All individuals were identified to species level and all species names were cross-referenced to match the accepted name provided by GBIF (*GBIF: The Global Biodiversity Information Facility*, 2024). Overall, the extensive sampling resulted in 1,374 species in 712 genera, 148 families, and 13 orders (Fig. 1). All

samples are stored at the Institute of Ecology and Evolution at the University Jena (Germany).

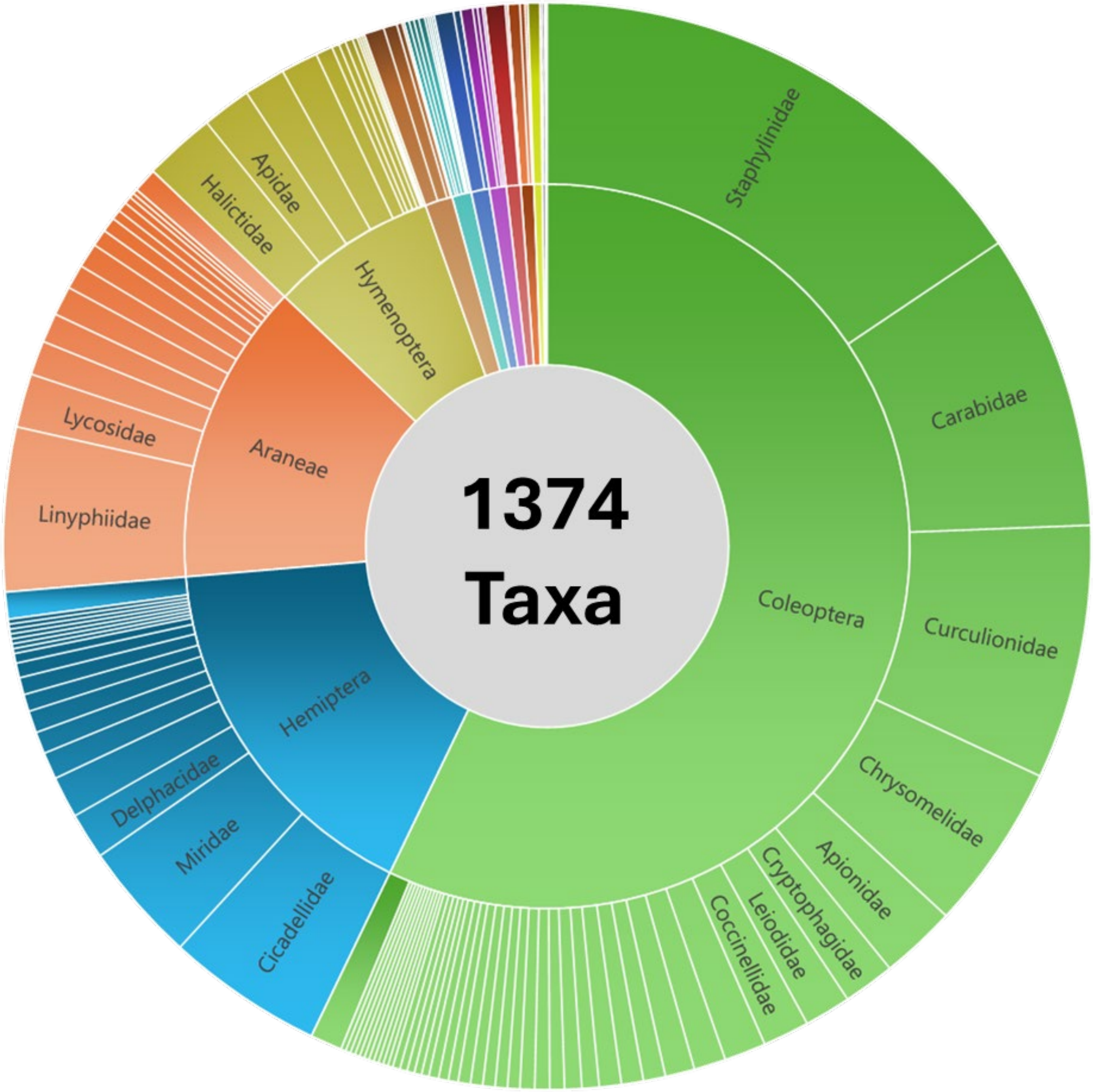

Figure 1: Illustration of coverage of trait information with two levels of categorization. The inner circle represents the level of orders, while the outer circle subdivides each order at the level of families. The size of each segment in both circles corresponds to the relative proportion of species with trait information within that order or family.

Research methods

An official permit was granted by the Environmental Protection Department of the city of Jena for the collection of invertebrates under species protection law.

a) Trait definition and assignment rules

Table 1: Description and classification of the arthropod traits.

Name	**Description**
Feeding guild (categorical)	Describes the type of food, which is consumed by the arthropod. They are divided into taxa that primarily feed on plant material (herbivores), animals (predators), dead organic matter (detritivores), and those that consume more than one resource type (omnivores).
Feeding source (categorical)	Gives information about the type of material which is consumed by the arthropod species. Taxa either consume plant material (leaves, stems, roots), plant sap (phloem, xylem), nectar and/ or pollen, animals, dead animals, dead plant material, feces or mycelium. For omnivores and detritivores combinations of the above listed categories can occur if both sources are equally consumed. However, if an omnivorous species predominantly feeds on one type of food, only that primary source is specified.
Food acquisition (categorical)	Describes how arthropods obtain their food. They can either passively catch their food (passive; web building spiders) or parasite other animals (parasitizing; e.g. beetles that parasite ant larvae). All other arthropods actively search for their food (active; e.g. ground beetles, leaf hoppers).

Feeding mode (categorical)	Describes how taxa process their food. A distinction is made between sucking, chewing and extraintestinal feeding consumers.
Food specialization (categorical)	Describes the range of food, which is consumed by the arthropod. Taxa are either monophagous (food resources belong to only one genus), oligophagous (food resources belong to one plant family or one order of animals) or polyphagous (food resources belong to more than one plant family or animal order). Detritivores are defined as monophagous or polyphagous if they consume only one or more sources of dead organic matter (see feeding source for all options), respectively.
Stratum (categorical)	Describes the habitat layer inhabited by the taxa. Taxa are classified into species living on the ground, the herb and tree layer, and species that are associated to animal nests, or water. Taxa can also inhabit multiple habitat types and move between layers. Parasitoids are assigned to the habitat layer corresponding to their respective hosts.
Aerial mobility (categorical)	The mobility of taxa is grouped into four categories, depending on their way and ability to fly. Taxa are either unable to fly, passive flyer (e.g. ballooning), active flyer during dispersion or mating, or active flyer during their entire adult stage.
Body mass (wet weight in mg)	Body mass was calculated based on body length values using the equations published in Sohlström *et al.* (2018).
Body length (mm)	For body length, mean lengths of adult males and females, generated from literature, were averaged..

b) Sources of the arthropod trait data

Traits for all species listed in Table 1 were generated from literature sources. When several sources had different trait information for the same species, we decided for the trait label with the highest reliability. Reliability of data sources were weighted as followed (from highest to lowest): 1) studies on the focal species, 2) books for species identification, 3) online databases, and 4) other online sources. For each species, the sources from which trait data originate are given. The reference numbers given in the data table refer to the references listed in Table 2.

Table 2: List of references used for generating the trait data

Index number and reference
1. Amiet, F. *et al.* (2001) *Apidae 3: Halictus, Lasioglossum. Fauna Helvetica 6*. Neuchâtel: Info fauna CSCF & SEG.
2. Amiet, F. *et al.* (2004) *Apidae 4: Anthidum, Chelostoma, Coelioxys, Dioxys, Heriades, Lithurgus, Megachile, Osmia, Stelis*. *Fauna Helvetica 9.* Neuchâtel: Info fauna CSCF & SEG.
3. Amiet, F. *et al.* (2008) *Apidae 5: Ammobates, Ammobatoides, Anthophora, Biastes, Ceratina, Dasypoda, Epeoloides, Epeolus, Eucera, Macropis, Melecta, Melitta, Nomada, Pasites, Tetralonia, Thyreus, Xylocopa*. *Fauna Helvetica 20*. Neuchâtel: Info fauna CSCF & SEG.
4. Amiet, F. *et al.* (2010) *Apidae 6: Andrena, Melitturga, Panurginus, Panurgus. Fauna Helvetica 26*. Neuchâtel: Info fauna CSCF & SEG.

5. Amiet, F., Müller, A. and Neumeyer, R. (2014) *Apidae 2: Colletes, Dufourea, Hylaeus, Nomia, Nomioides, Rhophitoides, Rophites, Sphecodes, Systropha*. *Fauna Helvetica 4*. Neuchâtel: Info fauna CSCF & SEG.

6. Amiet, F., Müller, A. and Praz, C. (2017) *Apidae 1: Allgemeiner Teil, Gattungen, Apis, Bombus*. *Fauna Helvetica 29*. Neuchâtel: Info fauna CSCF & SEG.

7. Anderson, R. and McFarren, D. (2023) *The Ground Beetles of Ireland*. Available at: http://www.habitas.org.uk/groundbeetles/ (Accessed: 24 June 2024).

8. Arriaga-Varela, E., Seidel, M. and Fikacek, M. (2018) 'A new genus of coprophagous water scavenger beetle from Africa (Coleoptera, Hydrophilidae, Sphaeridiinae, Megasternini) with a discussion on the Cercyon subgenus Acycreon', *African Invertebrates*, 59(1), pp. 1–23. Available at: https://doi.org/10.3897/AfrInvertebr.59.14621.

9. Assing, V. (2007) 'On the Aleocharini of Turkey, with notes on some species from adjacent regions (Coleoptera: Staphylinidae, Aleocharinae).', *Contributions to Entomology*, 57(1), pp. 177–209. Available at: https://doi.org/10.21248/contrib.entomol.57.1.177-209.

10. Assing, V. (2009) 'A revision of Ceranota Stephens, subgenus of Aleochara Gravenhorst (Coleoptera: Staphylinidae: Aleocharinae).', *Contributions to Entomology*, 59(2), pp. 355–422. Available at: https://doi.org/10.21248/contrib.entomol.59.2.355-422.

11. Assing, V. (2018) 'Revision of the Cousya species of the West Palaearctic Region (Coleoptera: Staphylinidae: Aleocharinae)', *Integrative Systematics: Stuttgart Contributions to Natural History*, 1(1), pp. 79–127. Available at: https://doi.org/10.18476/insy.v01.a9.

12. Assing, V. and Wunderle, P. (2008) 'On the Alevonota species of the Western Palaearctic region (Coleoptera: Staphylinidae: Aleocharinae: Athetini).', *Contributions to Entomology*, 58(1), pp. 145–189. Available at: https://doi.org/10.21248/contrib.entomol.58.1.145-189.

13. Avidal, R. (2024) *Insektarium*. Available at: https://insektarium.net/ (Accessed: 25 June 2024).

14. Balalaikins, M. and Bukejs, A. (2011) '*Otiorhynchus smreczynskii* (Coleoptera: Curculionidae) – a new to Estonia and Lithuania weevil species with notes on its occurrence and bionomy in the Eastern Baltic region', *Acta Zoologica Lituanica*, 21(4), pp. 263–267. Available at: https://doi.org/10.2478/v10043-011-0032-0.

15. Bantock, T. and Botting, J. (2022) *British Bugs Home - An online identification guide to UK Hemiptera*. Available at: http://www.britishbugs.org.uk/ (Accessed: 25 June 2024).

16. Bashir, A. *et al.* (2019) 'Taxonomic studies of subfamily Deltocephalinae and Typhlocybinae (Hemiptera: Cicadellidae) from District Faisalabad Punjab Pakistan with a key based on their Morphological Characters', *International Journal of Agriculture & Environmental Science*, 6(6), pp. 50–57. Available at: https://doi.org/10.14445/23942568/IJAES-V6I6P108.

17. Baulechner, D. *et al.* (2020) 'Convergent evolution of specialized generalists: Implications for phylogenetic and functional diversity of carabid feeding groups', *Ecology and Evolution*. 10(20), pp. 11100–11110. Available at: https://doi.org/10.1002/ece3.6746.

18. Baur, B., Gilgado, J. D. and Coray, A. (2023) ‘Prey handling and feeding habits of the snail predator Licinus depressus (Coleoptera, Carabidae)’, *Alpine Entomology*, 7(1), pp. 63–68. Available at: https://doi.org/10.3897/alpento.7.103164.

19. *Bees Wasps & Ants Recording Society* (2024). Available at: https://bwars.com/ (Accessed: 25 June 2024).

20. Bellmann, H. (2010) *Der Kosmos-Spinnenführer: über 400 Arten Europas*. Stuttgart: Kosmos.

21. Benisch, C. (2023) *Käfer (Coleoptera) der deutschen Käferfauna*. Available at: https://www.kerbtier.de/ (Accessed: 25 June 2024).

22. Biedermann, R. and Niedringhaus, R. (2004) *Die Zikaden Deutschlands. Bestimmungstafeln für alle Arten*. Scheeßel: WABV.

23. Biological Records Centre (2024) *UK Beetle Recording*. Available at: https://www.coleoptera.org.uk/home (Accessed: 25 June 2024).

24. Blandenier, G. (2009) ‘Ballooning of spiders (Araneae) in Switzerland: General Results from an Eleven-Year Survey’, *Arachnology*, 14(7), pp. 308–316. Available at: https://doi.org/10.13156/arac.2009.14.7.308.

25. Bleich, O., Gürlich, S. and Köhler, F. (2024) *Coleoptera Europaea - Verzeichnis und Verbreitungsatlas der Käfer Deutschlands*. Available at: http://www.coleoweb.de/ (Accessed: 25 June 2024).

26. Bouharroud, R. *et al.* (2019) ‘New record and predatory activity of Hyperaspis campestris (Herbst 1783)(Coleoptera: Coccinellidae) on Dactylopius opuntiae (Hemiptera: Dactylopiidae) in Morocco’, *Entomological News*, 128(2), pp. 156–160. Available at: https://doi.org/10.3157/021.128.0202.

27. British Arachnological Society (2024) *Spider and Harvestman Recording Scheme website*. Available at: https://srs.britishspiders.org.uk/portal.php (Accessed: 25 June 2024).

28. Byk, A. and Minkina, Ł. (2014) 'Rhyssemus puncticollis Brown, 1929 (Coleoptera: Scarabaeidae: Aphodiinae: Psammodiini): a new record for the fauna of Poland and Latvia', *The Coleopterists Bulletin*, 68(3), pp. 377–383. Available at: https://doi.org/10.1649/072.068.0305.

29. Campbell, J. (1979) 'A revision of the genus Tachyporus Gravenhorst (Coleoptera: Staphylinidae) of North and Central America', *Memoirs of the Entomological Society of Canada*, 111(S109), pp. 1–95. Available at: https://doi.org/10.4039/entm111109fv.

30. Castillo-Miralbés, M. (2011) 'Principales especies de Coleópteros necrófagos presentes en carroña de cerdos en la comarca de La Litera (Huesca)', *Graellsia*, 57(1), pp. 85–90. Available at: https://doi.org/10.3989/graellsia.2001.v57.i1.296.

31. Center for Invasive Species and Ecosystem Health. University of Georgia (2024) *Bugwood Wiki*. Available at: https://wiki.bugwood.org/Main_Page (Accessed: 25 June 2024).

32. Communications IFAS (2024) *Entomology and Nematology Department - University of Florida, Institute of Food and Agricultural Sciences - UF/IFAS*. Available at: https://entnemdept.ufl.edu/ (Accessed: 25 June 2024).

33. Decker, P. *et al.* (2024) *BODENTIER hoch 4 - Onlineportal mit App zum Erleben, Erkennen, Erfassen und Erforschen*. Available at: https://bodentierhochvier.de/ (Accessed: 24 June 2024).

34. Dittrich, A. and Helden, A. (2020) ‘Can monophagous specialists mediate host plant choices in generalist planthoppers (Hemiptera: Delphacidae)?’, *Ecological Entomology*, 45(6), pp. 1509–1512. Available at: https://doi.org/10.1111/een.12929.

35. Dollfuss, H. (1991) ‘Bestimmungsschlüssel der Grabwespen Nord-und Zentraleuropas’, *Stapfia*, 24(1), pp. 1–247.

36. Ellis, W. N. (2024) *Plant Parasites of Europe – leafminers, galls and fungi*. Available at: https://bladmineerders.nl/ (Accessed: 24 June 2024).

37. *Encyclopedia of Life* (2024). Available at: https://eol.org/ (Accessed: 25 June 2024).

38. Esser, J. (2005) ‘Volvariella gloiocephala ein Entwicklungspilz von Atomaria puncticollis THOMSON, 1868? (Coleoptera, Cryptophagidae)’, *Mitteilungen der Arbeitsgemeinschaft Rheinischer Koleopterologen*, 15(1–2), pp. 3–4.

39. Felke, M. (2024) *Institut für Schädlingskunde*. Available at: https://schaedlingskunde.de/ (Accessed: 24 June 2024).

40. Freude, H. *et al.* (1964) *Die Käfer Mitteleuropas*. Krefeld: Goecke & Evers.

41. Friedrich, M. (2024) *Insekten, Spinnen und andere Gliederfüßer - arthropodafotos.de*. Available at: https://arthropodafotos.de/ (Accessed: 25 June 2024).

42. Funk, W. (2024) *Insektenbox*. Available at: http://www.insektenbox.de/ (Accessed: 24 June 2024).

43. *GBIF: The Global Biodiversity Information Facility* (2024). Available at: https://www.gbif.org/ (Accessed: 24 June 2024).

44. Glotov, S. *et al.* (2022) ‘A review of species of the genus Mocyta (Coleoptera, Staphylinidae) in Ukraine’, *Biosystems Diversity*, 30(3), pp. 234–243. Available at: https://doi.org/10.15421/012225.

45. Gossner, M. M. *et al.* (2015) ‘A summary of eight traits of Coleoptera, Hemiptera, Orthoptera and Araneae, occurring in grasslands in Germany’, *Scientific Data*, 2(1), pp. 1–9. Available at: https://doi.org/10.1038/sdata.2015.13.

46. Green, A. J. (2021) *The Sawflies (Symphyta) of Britain and Ireland*. Available at: https://www.sawflies.org.uk/ (Accessed: 25 June 2024).

47. Harz, K. (1969) *Die Orthopteren Europas / The Orthoptera of Europe. Volume I*. The Hague: Dr. W. Junk Publishers.

48. Harz, K. (1975) *Die Orthopteren Europas / The Orthoptera of Europe. Volume II*. The Hague: Dr. W. Junk Publishers.

49. Haselböck, A. (2024) *Naturspaziergang: Naturlexikon - Naturfotografie*. Available at: http://www.naturspaziergang.de/index.htm (Accessed: 24 June 2024).

50. Heijerman, T. (1984) ‘Omiamima mollina (Boheman) in the Netherlands, with notes on habitat and phenology (Coleoptera: Curculionidae)’, *Entomologische Berichten*, 44(11), pp. 170–173.

51. Herger, P. (2005) ‘Zur Insektenfauna des Flachmoores Wauwilermoos, 498 m, Kanton Luzern. III. Coleoptera 1 (Käfer)’, *Entomologische Berichte Luzern*, 53(1), pp. 1–20.

52. Hines, J. *et al.* (2019) ‘A meta food web for invertebrate species collected in a European grassland’, *Ecology*, 100(6), e02679. Available at: https://doi.org/10.1002/ecy.2679.

53. Hlaváč, P. and Tomáš, J. (2009) ‘A revision of the genus Zyras (Zyras) Stephens, 1835 (Coleoptera, Staphylinidae, Aleocharinae). I. Current classification status and the redefinition of the genus’, *ZooKeys*, 29(1), pp. 49–71. Available at: https://doi.org/10.3897/zookeys.29.218.

54. Kania, G. and Kłapeć, T. (2012) ‘Seasonal activity of millipedes (Diplopoda)--their economic and medical significance’, *Annals of Agricultural and Environmental Medicine*, 19(4), pp. 646–650.

55. Kuřavová, K., Šipoš, J. and Kocarek, P. (2020) ‘Energy balance of food in a detrito-bryophagous groundhopper (Orthoptera: Tetrigidae)’, *PeerJ*, 8(1), e9603. Available at: https://doi.org/10.7717/peerj.9603.

56. Larson, S. G. and Gígja, G. (1959) ‘Coleoptera 1. Synopsis’, *The Zoology of Iceland*, 3.

57. Lindsey, J. K. (2024) *Commanster*. Available at: https://www.commanster.eu/ (Accessed: 25 June 2024).

58. Liz, B. (1999) *Klucze do oznaczania owadów Polski. Część XVIII. Pluskwiaki różnoskrzydłe – Heteroptera, zeszyt 8. Prześwietlikowate – Tingidae*. Toruń: Polskie Towarzystwo Entomologiczne.

59. Lompe, A. (2024) *Käfer Europas*. Available at: https://coleonet.de/coleo/index.htm (Accessed: 24 June 2024).

60. Majka, C., Colin, J. and Langor, D. (2010) ‘Contributions towards an understanding of the Atomariinae (Coleoptera, Cryptophagidae) of Atlantic Canada’, *ZooKeys*, 35(1), pp. 13–35. Available at: https://doi.org/10.3897/zookeys.35.318.

61. Martin, H.-J. (2000) *Wildbienen: Solitärbienen & Hummeln - Biologie, Arten, Schutz*. Available at: https://www.wildbienen.de/ (Accessed: 24 June 2024).

62. Maus, C. and Ausmeier, F. (1999) ‘Aleochara (Heterochara) spissicornis Erichson, 1839 in Südbaden–ein Wiederfund für Südwestdeutschland nach über 50 Jahren (Coleoptera: Staphilinidae)’, *Mitteilungen des Entomologischen Vereins Stuttgart*, 34(1), pp. 135–138.

63. McElrath, T. C., Boyd, O. F. and McHugh, J. V. (2016) ‘MonotomidGen – A matrix-based interactive key to the New World genera of Monotomidae (Coleoptera, Cucujoidea)’, *ZooKeys*, 634(1), pp. 47–55. Available at: https://doi.org/10.3897/zookeys.634.9857.

64. *NatureSpot* (2024). Available at: https://www.naturespot.org.uk/ (Accessed: 24 June 2024).

65. Nentwig, W. *et al.* (2024) ‘Spiders of Europe’. University of Bern. Available at: https://doi.org/10.24436/1.

66. Noort, S. van (2024) *WaspWeb - Hymenoptera of the World*. Available at: https://www.waspweb.org// (Accessed: 24 June 2024).

67. Pearce, E. J. (1957) ‘Handbooks for identification of British insects. Vol. IV, Part 9. Coleoptera (Pselaphidae).’, *Royal Entomological Society’,* 4(9), pp. 1–32.

68. Pendleton, T. and Pendleton, D. (2024) *Eakring Birds*. Available at: http://www.eakringbirds.com/ (Accessed: 25 June 2024).

69. *Picture Insect - Online insect encyclopedia and insect identifier* (2024). Available at: https://pictureinsect.com (Accessed: 25 June 2024).

70. Rennwald, S. (2024) *Nafoku: Natur- und Foto-Kunst*. Available at: https://nafoku.de/ (Accessed: 25 June 2024).

71. Riedel, J. (2023) *Digital-Nature - Eine Symbiose aus Natur, Wissenschaft und Kunst*. Available at: https://www.digital-nature.de/ (Accessed: 25 June 2024).

72. Rutschmann, F. and Roesti, C. (2024) *Orthoptera.ch*. Available at: https://www.orthoptera.ch/ (Accessed: 25 June 2024).

73. Salnitska, M. and Solodovnikov, A. (2019) ‘Rove beetles of the genus Quedius (Coleoptera, Staphylinidae) of Russia: a key to species and annotated catalogue’, *ZooKeys*, 847(1), p. 1–100. Available at: https://doi.org/10.3897/zookeys.847.34049.

74. Schatz, I. (2009) ‘Staphylinidae) in den Ahrauen bei Bruneck–Neumeldung für Südtirol und Italien vom Tag der Artenvielfalt 2009’, *Grendleriana*, 9(1), pp. 281–282.

75. Schawaller, W. (2018) ‘Taxonomy and distribution of the species of the genus Myrmechixenus Chevrolat, 1835 (Tenebrionidae: Diaperinae)’, *Integrative Systematics: Stuttgart Contributions to Natural History*, 1(1), pp. 1–6.

76. Schmidt, U. (2024) *Käfer der Welt*. Available at: https://www.kaefer-der-welt.de/ (Accessed: 24 June 2024).

77. Schülke, M. and Kocian, M. (2000) ‘Revision der Artgruppe des Mycetoperus nigricollis STEPHENS, 1835 (Coleoptera, Staphylinidae, Tachyporinae)’, *Entomologische Blätter für Biologie und Systematik der Käfer*, 96(2), pp. 80–126.

78. Sebesta, M. (2024) *Ameisenhaltung*. Available at: https://www.ameisenhaltung.de/ (Accessed: 24 June 2024).

79. Semenov, V. B. (2007) ‘Staphilinidy podsemeistva Aleocharinae (Coleoptera: Staphylinidae) Moskovskoi oblasti. Chastʹ 1. Triby Deinopsini–Athetini [The staphylinid beetles subfamily Aleocharinae (Coleoptera: Staphylinidae) of the Moscow Area. Part 1. The tribes Deinopsini–Athetini]’, *Eversmannia*, pp. 11–52, 24–52.

80. Smetana, A. (1995) *Rove beetles of the subtribe Philonthina of America North of Mexico (Coleoptera: Staphylinidae) classification, phylogeny and taxonomic revision.* Gainesville: Associated Publishers.

81. Solari, C. and Rivelle, M. (2024) *SpeciesConnect*. Available at: https://speciesconnect.com (Accessed: 25 June 2024).

82. Staniec, B. and Pietrykowska-Tudruj, E. (2009) ‘Immature stages of Rabigus tenuis (Fabricius, 1792)(Coleoptera, Staphylinidae, Staphylininae) with observation on its biology and taxonomic comments’, *Belgian Journal of Zoology*, 139(1), pp. 22–39.

83. Steiner, A. (2024) *Natur in NRW*. Axel Steiner, axelsteiner@web.de. Available at: https://www.natur-in-nrw.de/ (Accessed: 24 June 2024).

84. Stöffler, M. (2008) ‘Zur Biologie myrmekophiler Kurzflügelkäfer der Gattung Pella (Coleoptera, Staphylinidae) in Baden-Württemberg unter besonderer Berücksichtigung von chemischer Verteidigung und Mimikry’, *Jahreshefte der Gesellschaft für Naturkunde in Württemberg*, 164(1), pp. 171–195.

85. Stresemann, E. (2011) *Stresemann-Exkursionsfauna von Deutschland, Band 2: Wirbellose: Insekten*. Heidelberg: Springer-Verlag.

86. Telfer, M. G. (2018) ‘The status and distribution of the ground beetle Harpalus melancholicus at Stackpole Warren in 2017’, *NRW Evidence Report,* 247, pp. 1–26, Natural Resources Wales, Bangor.

87. The Lucid Team (2024) *Lucidcentral*. Available at: https://www.lucidcentral.org/ (Accessed: 25 June 2024).

88. Tymann, G. (2024) *Wanzen-im-Ruhrgebiet*. Available at: https://www.wanzen-im-ruhrgebiet.de/ (Accessed: 24 June 2024).

89. *UK Beetles* (2023). Available at: https://www.ukbeetles.co.uk (Accessed: 24 June 2024).

90. Ulmer, E. (2024) *Wildbienenwelt.de – Das große Wildbienenportal*. Available at: https://www.wildbienenwelt.de (Accessed: 24 June 2024).

91. VanDyk, J. (2024) *BugGuide*. Available at: https://bugguide.net/node/view/15740 (Accessed: 24 June 2024).

92. Varga, O. and Kostro-Ambroziak, A. (2023) 'New Records of Poemeniinae and Xoridinae (Hymenoptera, Ichneumonidae) from Ukraine and Poland, with Corrections to the Ukrainian Checklist', *Zoodiversity*, 57(3), pp. 277–282. Available at: https://doi.org/10.15407/zoo2023.03.277.

93. Varvara, M. and Apostol, E. (2008) 'Diversity and the main ecological requirements of the epigeic species of Carabidae (Coleoptera, Carabidae) in the sunflower ecosystem, Broscăuţi (Botoşani County)', *Biologie Animala*, 54(1), pp. 1–9.

94. Vilisics, F., Sólymos, P. and Hornung, E. (2007) 'A preliminary study on habitat features and associated terrestrial isopod species', *Contributions to Soil Zoology in Central Europe II*, pp. 195–199.

95. Wachmann, E., Melber, A. and Deckert, J. (2004) *Wanzen Bd. 2 Cimicomorpha: Microphysidae (Flechtenwanzen), Miridae (Weichwanzen)*. Keltern: Goecke & Evers.

96. Wachmann, E., Melber, A. and Deckert, J. (2006) *Wanzen Bd. 1 Dipsocoromorpha, Nepomorpha, Gerromorpha, Leptopodomorpha, Cimicomorpha (Teil 1)*. Keltern: Goecke & Evers.

97. Wachmann, E., Melber, A. and Deckert, J. (2007) *Wanzen Bd. 3 Pentatomomorpha : 1, Aradidae, Lygaeidae, Piesmatidae, Berytidae, Pyrrhocoridae, Alydidae, Coreidae, Rhopalidae, Stenocephalidae*. Keltern: Goecke & Evers.

98. Wachmann, E., Melber, A. and Deckert, J. (2008) *Wanzen Bd. 4 Pentatomomorpha : 2, Pentatomoidea: Cydnidae, Thyreocoridae, Plataspidae, Acanthosomatidae, Scutelleridae, Pentatomidae*. Keltern: Goecke & Evers.

99. Wahl, D. B. and Gauld, I. D. (2024) *Genera Ichneumonorum Nearcticae*. Available at: http://www.amentinst.org/GIN/ (Accessed: 25 June 2024).

100. Wanat, M. (2011) ‘Biology and distribution of Lixus punctiventris Boheman, 1835 (Coleoptera, Curculionidae) in Poland’, *Weevil News*, 64(1), pp. 1–5.

101. Wiki der Arachnologischen Gesellschaft e. V.-Bearbeiter (2024) *Wiki der Arachnologischen Gesellschaft e. V.* Available at: https://wiki.arages.de/index.php?title=Hauptseite (Accessed: 24 June 2024).

102. Wikipedia contributors (2024) *Wikipedia: The Free Encyclopedia*. Available at: https://de.wikipedia.org/w/index.php?title=Wikipedia:Hauptseite&oldid=243938148 (Accessed: 24 June 2024).

103. Wilson, M. R. and Turner, J. A. (2021) *Insect Vectors of Plant Disease*. Available at: https://insectvectors.science/ (Accessed: 25 June 2024).

104. Witt, R. (1998) *Wespen: beobachten, bestimmen*. Augsburg: Naturbuch-Verlag.

105. Zahniser, J. N. (2015) *The leafhopper subfamily Deltocephalinae*. Available at: http://zahniser.speciesfile.org/ (Accessed: 25 June 2024).

106. *Zwergmaskenzikade* (2024) *Blühendes Österreich*. Available at: https://www.bluehendesoesterreich.at/naturlexikon/zwergmaskenzikade (Accessed: 25 June 2024).

Class III. Data set status and accessibility

A. Status

1. **Latest update:** December 2024
2. **Latest archive date:** Date of last data set archival

3. **Metadata status:** Metadata is current and up to date (last updated: December 2024)
4. **Data verification:** All species names were cross-referenced with the online database GBIF (*GBIF: The Global Biodiversity Information Facility*, 2024) to check for synonyms and any changes in species names across sampling campaigns. However, species names may change in the future (last updated: December 2024). Multiple sources were considered for each taxon to provide robust information on their ecology, behavior, and body length. While the reliability of these traits depends on careful and systematic data collection, some traits are derived from field observations and assumptions that may not fully capture the species' complete ecology. Additionally, traits can depend on ecological and environmental contexts and often exhibit underlying intraspecific variability.

B. Accessibility

1. **Storage location and medium:** Data are provided as supporting information with the submission and archived in the following repository: https://doi.org/10.25829/Q570-JR82 (Bröcher *et al.* 2025).
2. **Contact persons:**

Maximilian Bröcher: Institute of Ecology and Evolution, University Jena, Dornburger Strasse 159, D-07743 Jena, Germany. E-mail: maximilian.broecher@uni-jena.de

Anne Ebeling: Institute of Ecology and Evolution, University Jena, Dornburger Strasse 159, D-07743 Jena, Germany. E-mail: anne.ebeling@uni-jena.de

3. **Copyright restrictions:** None

4. **Proprietary restrictions:** None

5. **Costs:** None

Class IV. Data structural descriptors

A. Data set file

The dataset is downloadable as a single zipped archive, DataS1.zip (33KB), which contains the following file stored as comma-separated values (.csv).

species_traits_JE.csv (175KB): Species traits and literature information. The species trait data contains the species name, taxonomic identity, species traits and literature references of the 1,374 arthropod species collected by pitfall and suction sampling in the Jena Experiment. For header information see Table 3.

B. Variable information

Table 3: Overview of all variables in species traits data set: "species_traits_JE.csv".

Taxa	Species scientific name (Genus, Species)
Genus	Taxonomic resolution Genus
Family	Taxonomic resolution Family
Order	Taxonomic resolution Order
Class	Taxonomic resolution Class

Feeding guild	The type of food, which is consumed by the consumer (detritivores, herbivores, omnivores, predators)
Feeding source	The type of material which is consumed by the arthropod species (plant material, plant sap, nectar_pollen, animals, dead animals, dead plant material, feces, mycelium)
Food acquisition	Describes how consumers obtain their food (active, parasitizing, passive)
Feeding mode	Describes how taxa process their food (extraintestinal, chewing, sucking)
Food specialization	The range of food, which is consumed by the arthropod species (m = monophagous, o = oligophagous, p = polyphagous)
Stratum	The habitat layer inhabited by the taxa (g = ground, h = herb, n = animal nests, t = tree, w = water and all possible combinations of g, h, n, t and w separated by slash)
Aerial mobility	The mobility of taxa (a = unable to fly, b = passive flyer, c = active flyer during dispersion or mating, d = active flyer during their entire adult stage)
Body mass	Body mass in mg (numeric)
Body length	Body size in mm (numeric)
Reference	Literature reference for species traits. The numbers refer to references listed in Table 2.

Missing values are coded as NA and occur if for a species no trait information was found.

Class V. Supplemental descriptors

A. Data acquisition

B. Quality assurance/quality control procedures:

C. Related materials:

D. Computer programs and data-processing algorithms:

E. Archiving

F. Publications and results: Electronic reprints, lists of publications resulting from or related to the study, graphical/statistical data representations, etc.

Barnes, A. D. et al. (2020) 'Biodiversity enhances the multitrophic control of arthropod herbivory', *Science Advances*, 6(45), eabb6603. Available at: https://doi.org/10.1126/sciadv.abb6603.

Bröcher, M. et al. (2024) 'The positive plant diversity/consumer relationship is independent of grassland age', *Basic and Applied Ecology*, 76(1), pp. 58–68. Available at: https://doi.org/10.1016/j.baae.2024.02.006.

Buzhdygan, O. Y. et al. (2020) 'Biodiversity increases multitrophic energy use efficiency, flow and storage in grasslands', *Nature Ecology & Evolution*, 4(3), pp. 393–405. Available at: https://doi.org/10.1038/s41559-020-1123-8.

Ebeling, A. et al. (2018) 'Plant diversity effects on arthropods and arthropod-dependent ecosystem functions in a biodiversity experiment', *Basic and Applied Ecology*, 26(1), pp. 50–63. Available at: https://doi.org/10.1016/j.baae.2017.09.014.

Giling, D. P. et al. (2019) 'Plant diversity alters the representation of motifs in food webs', *Nature Communications*, 10(1), 1226. Available at: https://doi.org/10.1038/s41467-019-08856-0.

Hines, J. et al. (2019) 'A meta food web for invertebrate species collected in a European grassland', *Ecology*, 100(6), e02679. Available at: https://doi.org/10.1002/ecy.2679.

History of data set usage

Literature Citations

Bello, F. de *et al.* (2021) 'Functional trait effects on ecosystem stability: assembling the jigsaw puzzle', *Trends in Ecology & Evolution*, 36(9), pp. 822–836. Available at: https://doi.org/10.1016/j.tree.2021.05.001.

Brose, U. *et al.* (2006) 'Consumer–Resource Body-Size Relationships in Natural Food Webs', *Ecology*, 87(10), pp. 2411–2417.Available at: https://doi.org/10.1890/0012-9658(2006)87[2411:CBRINF]2.0.CO;2.

Bröcher, M., Meyer, S., Leher, A. G., & Ebeling, A. (2025). Data from: Ecological traits for 1,374 arthropod species collected in a German grassland [Data set]. The Jena Experiment Information System. https://doi.org/10.25829/Q570-JR82

Díaz, S. and Cabido, M. (2001) 'Vive la difference: plant functional diversity matters to ecosystem processes', *Trends in Ecology and Evolution*, 16(11), pp. 646–655. Available at: https://doi.org/10.1016/S0169-5347(01)02283-2.

Ebeling, A. *et al.* (2014) 'A trait-based experimental approach to understand the mechanisms underlying biodiversity-ecosystem functioning relationships', *Basic and Applied Ecology*, 15(3), pp. 229–240. Available at: https://doi.org/10.1016/j.baae.2014.02.003.

Ellenberg, H. and Leuschner, C. (2010) *Vegetation Mitteleuropas mit den Alpen: in ökologischer, dynamischer und historischer Sicht*. 6th edn. Stuttgart: Eugen Ulmer.

GBIF: The Global Biodiversity Information Facility (2024). Available at: https://www.gbif.org/. (Accessed: 24 June 2024).

Huang, Y. *et al.* (2024) 'Enhanced stability of grassland soil temperature by plant diversity', *Nature Geoscience*, 17(1), pp. 44–50. Available at: https://doi.org/10.1038/s41561-023-01338-5.

Lavorel, S. *et al.* (2013) 'A novel framework for linking functional diversity of plants with other trophic levels for the quantification of ecosystem services', *Journal of Vegetation Science*, 24(5), pp. 942–948. Available at: https://doi.org/10.1111/jvs.12083.

Martínez-Núñez, C. *et al.* (2024) 'Land-use change in the past 40 years explains shifts in arthropod community traits', *Journal of Animal Ecology*, 93(5), pp. 540–553. Available at: https://doi.org/10.1111/1365-2656.14062.

McGill, B.J. *et al.* (2006) 'Rebuilding community ecology from functional traits', *Trends in Ecology & Evolution*, 21(4), pp. 178–185. Available at: https://doi.org/10.1016/j.tree.2006.02.002.

Noriega, J.A. *et al.* (2018) 'Research trends in ecosystem services provided by insects', *Basic and Applied Ecology*, 26, pp. 8–23. Available at: https://doi.org/10.1016/j.baae.2017.09.006.

Roscher, C. *et al.* (2004) 'The role of biodiversity for element cycling and trophic interactions: an experimental approach in a grassland community', *Basic and Applied Ecology*, 5(2), pp. 107–121. Available at: https://doi.org/10.1078/1439-1791-00216.

Schowalter, T.D., Noriega, J.A. and Tscharntke, T. (2018) 'Insect effects on ecosystem services—Introduction', *Basic and Applied Ecology*, 26, pp. 1–7. Available at: https://doi.org/10.1016/j.baae.2017.09.011.

Sohlström, E.H. *et al.* (2018) ‘Applying generalized allometric regressions to predict live body mass of tropical and temperate arthropods’, *Ecology and Evolution*, 8(24), pp. 12737–12749. Available at: https://doi.org/10.1002/ece3.4702.

Violle, C. *et al.* (2007) ‘Let the concept of trait be functional!’, *Oikos*, 116(5), pp. 882–892. Available at: https://doi.org/10.1111/j.2007.0030-1299.15559.x.

Wong, M.K.L., Guénard, B. and Lewis, O.T. (2019) ‘Trait-based ecology of terrestrial arthropods’, *Biological Reviews*, 94(3), pp. 999–1022. Available at: https://doi.org/10.1111/brv.12488.

Woodward, G. *et al.* (2005) ‘Body size in ecological networks’, *Trends in Ecology & Evolution*, 20(7), pp. 402–409. Available at: https://doi.org/10.1016/j.tree.2005.04.005.
